# Whole exome sequencing identifies a recurrent *RQCD1* P131L mutation in cutaneous melanoma

**DOI:** 10.18632/oncotarget.2747

**Published:** 2014-12-06

**Authors:** Stephen Q. Wong, Andreas Behren, Victoria J. Mar, Katherine Woods, Jason Li, Claire Martin, Karen E. Sheppard, Rory Wolfe, John Kelly, Jonathan Cebon, Alexander Dobrovic, Grant A. McArthur

**Affiliations:** ^1^ Division of Cancer Research, Peter MacCallum Cancer Centre, East Melbourne, Victoria, Australia; ^2^ Ludwig Institute for Cancer Research, Olivia Newton-John Cancer and Wellness Centre Heidelberg, Victoria, Australia; ^3^ School of Cancer Medicine, La Trobe University, Bundoora, Victoria, Australia; ^4^ Victorian Melanoma Service, Alfred Hospital, Prahran, Victoria, Australia; ^5^ Department of Epidemiology and Preventive Medicine, Monash University, Clayton, Victoria, Australia; ^6^ Department of Biochemistry and Molecular Biology, University of Melbourne, Parkville, Victoria, Australia; ^7^ Department of Pathology, University of Melbourne, Parkville, Victoria, Australia

**Keywords:** Melanoma, RQCD1, exome sequencing

## Abstract

Melanoma is often caused by mutations due to exposure to ultraviolet radiation. This study reports a recurrent somatic C > T change causing a P131L mutation in the *RQCD1* (Required for Cell Differentiation1 Homolog) gene identified through whole exome sequencing of 20 metastatic melanomas. Screening in 715 additional primary melanomas revealed a prevalence of ~4%. This represents the first reported recurrent mutation in a member of the CCR4-NOT complex in cancer. Compared to tumors without the mutation, the P131L mutant positive tumors were associated with increased thickness (*p* = 0.02), head and neck (*p* = 0.009) and upper limb (*p* = 0.03) location, lentigo maligna melanoma subtype (*p* = 0.02) and *BRAF* V600K (*p* = 0.04) but not V600E or *NRAS* codon 61 mutations. There was no association with nodal disease (*p* = 0.3). Mutually exclusive mutations of other members of the CCR4-NOT complex were found in ~20% of the TCGA melanoma dataset suggesting the complex may play an important role in melanoma biology. Mutant *RQCD1* was predicted to bind strongly to HLA-A0201 and HLA-Cw3 MHC1 complexes. From thirteen patients with mutant *RQCD1*, an anti-tumor CD8^+^ T cell response was observed from a single patient's peripheral blood mononuclear cell population stimulated with mutated peptide compared to wildtype indicating a neoantigen may be formed.

## INTRODUCTION

Melanoma is an aggressive form of skin cancer, with mutations in *BRAF* and *NRAS* genes occurring in ~50% and ~15% of tumors respectively. Exposure to ultraviolet (UV) radiation induces C > T nucleotide changes at dipyrimidine sites which are responsible for high mutation rates observed in melanomas [1], particularly those wild-type for both *BRAF* and *NRAS* [2].

In recent years, genomic sequencing studies of melanoma have uncovered mutations in multiple genes including *ERBB4* [3], *GRIN2A* [4] and *PREX2* [5]. Whilst most UV induced mutations are likely to be passenger events, others have been suggested to be involved in tumor development such as the activating *RAC1* P29S mutation that occurs in approximately 5% of melanoma cases [1, 6, 7]. Recently, noncoding C > T mutations have also been identified in the telomerase reverse transcriptase (*TERT*) promoter [8, 9], the *RPS27* 5′ untranslated region [10] and in the *BCL2L12* gene [11], with mutations potentially playing important roles in melanoma development and/or progression. Other biologically important mutations, particularly those associated with UV damage are still yet to be discovered.

Here we report the identification of a new recurrent mutation, P131L, in the *RQCD1* gene which encodes for the CCR4-NOT Transcription Complex Subunit 9; a highly conserved transcription (co)factor that plays a role in multiple biological processes including cellular differentiation and RNA processing [12].

## RESULTS

### Identification of a recurrent *RQCD1* P131L mutation in melanoma

To identify novel driver genes in melanoma, we screened for somatic mutations in 20 metastatic melanoma cell lines and paired matched blood DNA using exome sequencing [13]. The average sequencing depth was 152-fold (range from 84–287), and 94.4% (range from 87.4–97.9%) of the target regions were covered at least 10-fold (Supplementary Table 1).

**Figure 1:**
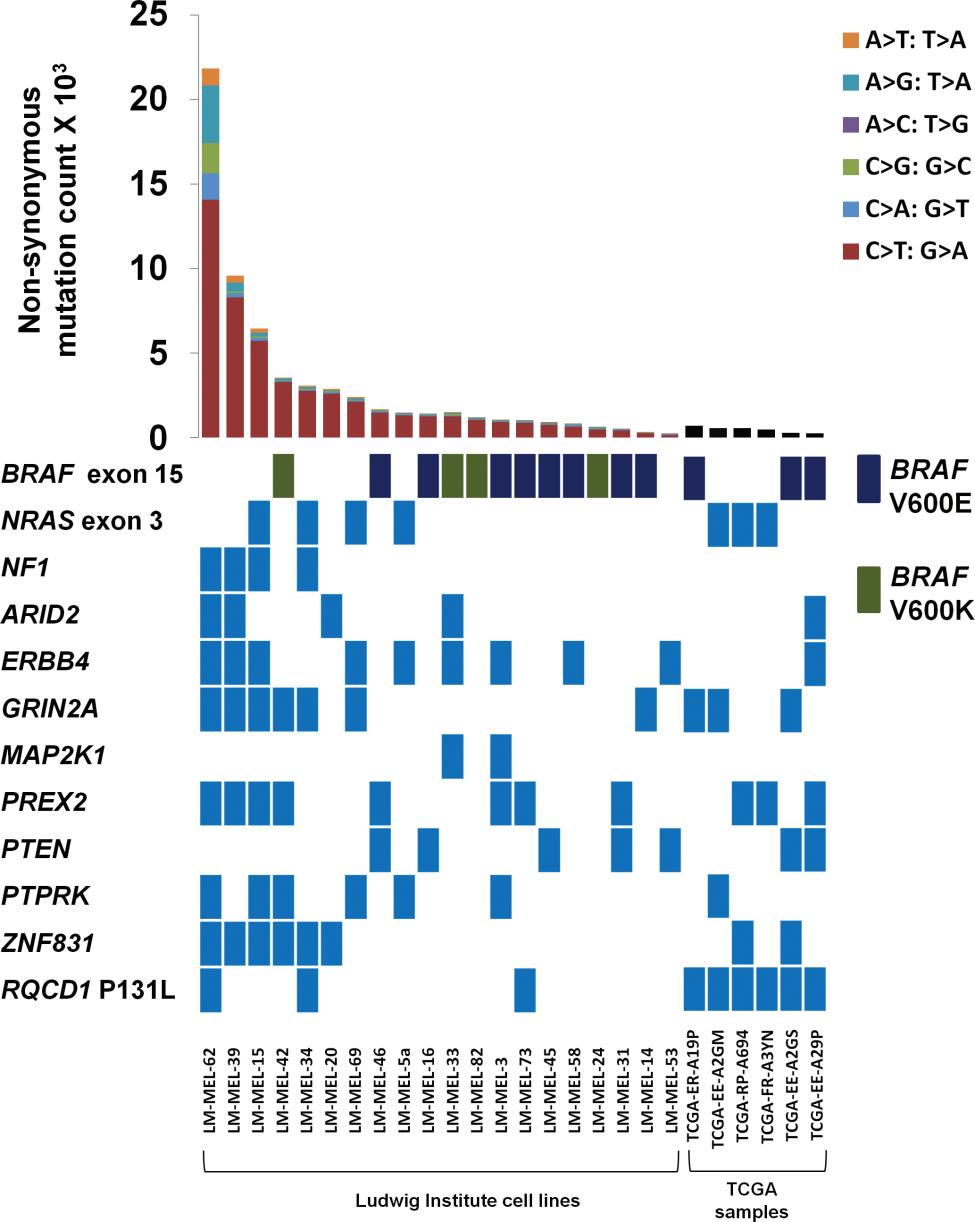
Mutational landscape of 20 metastatic melanomas Genetic landscape of 20 metastatic melanoma cell lines from the Ludwig institute and *RQCD1* P131L mutant melanomas from the TCGA dataset. Numbers of somatic non-synonymous mutations across matched melanoma samples are shown in the top bar graph with the type of nucleotide change indicated in the legend (Not shown for TCGA dataset). The mutational status of samples is indicated for classical melanoma genes including *BRAF*, *NRAS*, *NF1*, *PREX2, MAP2K1* and *PTEN* with a light blue coloured rectangle indicating the presence of at least one mutation in the gene. Only mutations in exon 15 of *BRAF*, exon 3 of *NRAS*, and the *RQCD1* P131L are shown. For *BRAF*, V600E, V600K are represented by a dark blue or green rectangle, respectively.

There were a total of 68,450 exomic somatic mutations in this dataset, with the average tumor displaying 3,422 (range from 269–26,493) somatic mutations. Consistent with UV damage, the tumors displayed a disproportionate level of C > T/G > A mutations which on average, accounted for 81% of all nucleotide changes (Figure 1). Recurrent non-synonymous mutations, including *BRAF* V600E (*n* = 8)/V600K (*n* = 4) were found as well as codon 61 mutations in *NRAS* (*n* = 4). Not unexpectedly, low mutation rates were associated with positive *BRAF* (*p* = 0.006) and *NRAS* mutation status (*p* = 0.04). While there was a trend for *BRAF/NRAS* wildtype tumors to be associated with high mutation loads, this did not reach statistical significance because of the small number of cases in this cohort (*p* = 0.14).

Other recurrent non-synonymous mutations identified included four cases of *ADAM7* G302E mutation and three cases of *PPP6C* R301C mutation. Mutations in these genes associated with melanoma have been previously been reported in other studies and these genes appear to function as tumor suppressor genes [14, 15] (Table 1). Other mutations in different regions of these genes were also identified in the 20 melanoma cell lines.

**Table 1:** Recurrent non-synonymous mutations from the discovery cohort of 20 metastatic melanoma cell lines Only recurrent mutations that were identified ≥ 3 times are shown

Number of samples with this variant	Gene Name	CDS position	Protein position	Amino acids
8	*BRAF*	1799	600	V/E
4	*ADAM7*	905	302	G/E
4	*BRAF*	1798_1799	600	V/K
3	*NRAS*	182	61	Q/R
3	*PCDHB8*	931	311	E/K
3	*PPP6C*	901	301	R/C
3	*RQCD1*	392	131	P/L
3	*VWA3B*	1073	358	V/G
3	*ZNF208*	3655	1219	H/Y

Three of the 20 melanoma cell lines had a hotspot P131L mutation in a highly conserved region of the *RQCD1* (required for cell differentiation) gene. Investigation of other melanoma genomic datasets revealed the *RQCD1* P131L in 2 of 147 cases (Krauthammer et al. [1]), 1/135 cases (Hodis et al. [6]), 2/34 cases (Mar et al. [2]), 10/489 cases (Dutton-Regester et al. [10]) and 6/279 (2.2%) in the TCGA provisional set (Figure 1). There was no evidence of this mutation in any other tumor type besides melanoma including other high mutation load cancers such as lung adenocarcinoma or head and neck squamous cell carcinoma.

In addition to the hotspot *RQCD1* mutation, we also identified recurrent mutations in the *PCDHB8* (E311K, *n* = 3)*, VWA3B* (V358G, *n* = 3) and *ZNF208* (H1219Y, *n* = 3) genes. Subsequent investigation of other melanoma genomic datasets could not confirm the recurrence of these mutations.

### Clinical features of *RQCD1* P131L mutant melanomas

**Figure 2:**
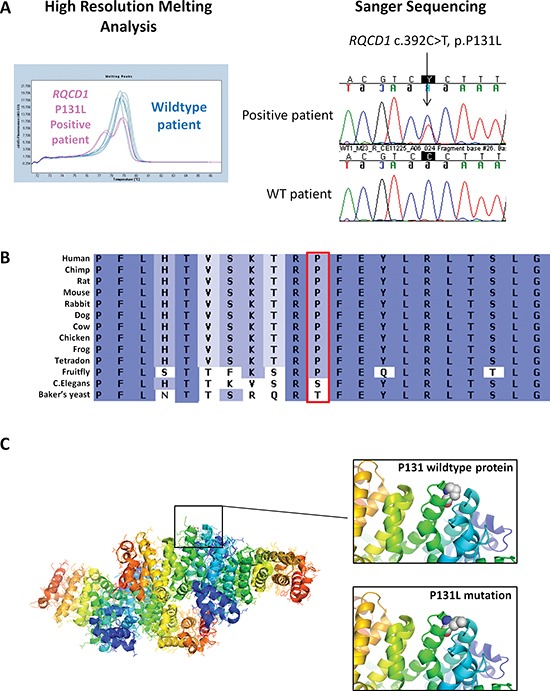
Recurrent hotspot *RQCD1* P131L mutation **(A)** Representative melting peak curve from a high resolution melting analysis of the *RQCD1* P131L region. Three wildtype patients are shown in blue with one *RQCD1* P131L positive sample shown in pink. Validation of aberrant melt curves from the high resolution melting analysis was performed using Sanger sequencing (Shown are sequencing chromatograms from a positive *RQCD1* P131L patient and a wildtype patient). **(B)** Conservation of *RQCD1* at the P131 residue across various species **(C)** Location of P131 residue on crystal structure of *RQCD1*. Zoomed view: Rendered spherical chemical structures of the wildtype proline at position 131 and mutant leucine residue. The protein crystal structure of the Human *RQCD1* was generated using PyMOL software 3.0.

We validated the *RQCD1* P131L mutations by screening an additional 715 cases of primary cutaneous melanoma by high resolution melting analysis (HRM) screening and Sanger sequencing of samples with heteroduplexes (Figure 2a). Of the 715 cases, 29 (4%) had the *RQCD1* P131L mutation. Eleven cases with the *RQCD1* mutation were female and 18 male (*p* = 0.7) (Table 2). The median age of patients with *RQCD1* mutant melanomas was 61 years compared to 58 years for patients without the mutation (*p* = 0.6).

**Table 2:** Associations between *RQCD1* mutation and other clinical and histological variables

	*RQCD1* mutant		*RQCD1* WT				
Continuous Variables	Median	[IQR]	Median	[IQR]	OR	95%CI	*p*-value
**Age (years)**	61	[51, 71]	58	[46, 68]	1.0	1.0, 1.0	0.6
**Thickness (mm)**	2.3	[1.2, 4.0]	1.4	[0.8, 2.7]	1.4	1.1, 1.8	0.02
**Mitotic rate (mm^2^)**	4	[1, 6]	2	[1, 6]	1.1	0.9, 1.3	0.3
**Categorical variables**	**n**	**%**	**n**	**%**	**OR**	**95%CI**	***p*-value**
Total cohort	29	4	686	96			
**Gender**							
Male	18	62	400	58			
Female	11	38	285	42	0.9	0.4, 1.8	0.7
**Site**							
T	3	10	207	32			
HN	11	38	134	21	5.7	1.6, 20.7	0.009
UL	10	34	168	26	4.1	1.1, 15.2	0.03
LL	5	17	124	19	2.8	0.7, 11.8	0.2
Other	0	0	17	3	-	-	-
**Subtype**							
SSM	12	43	386	60			
NM	10	36	168	26	1.9	0.8, 4.5	0.1
LMM	5	18	42	7	3.8	1.3, 11.4	0.02
Other	1	4	43	7	0.7	0.1, 5.9	0.8
**Ulceration**							
No	19	66	486	77			
Yes	10	34	145	23	1.8	0.8, 3.9	0.2
**Regression**							
No	20	80	407	71			
Yes	5	20	165	29	0.6	0.2, 1.7	0.3
**Presence of tumor infiltrating lymphocytes**							
No	12	46	253	49			
Yes	14	54	262	51	1.1	0.5, 2.5	0.8
**Solar elastosis**							
Non-SSD	9	41	125	43			
SSD	13	59	163	57	1.1	0.5, 2.7	0.8
**History of solar keratoses**							
No	10	45	346	57			
Yes	12	55	264	43	1.6	0.7, 3.7	0.3
**History of NMSC**							
No	17	71	478	72			
Yes	7	29	183	28	1.1	0.4, 2.6	0.2
**History of blistering sunburn**							
No	6	27	240	37			
Yes	16	73	410	63	1.6	0.6, 4.0	0.4

WT wild type; IQR interquartile range; SSM superficial spreading melanoma; NM nodular melanoma; LMM lentigo maligna melanoma; HN head and neck; UL upper limb; T trunk; LL lower limb; SSD severely sun-damaged skin; NMSC non-melanoma skin cancer.

Tumors harboring the *RQCD1* P131L mutation were significantly thicker than tumors without the mutation (median thickness 2.3 mm vs. 1.4 mm, OR = 1.4 for a doubling in mm thickness, 95%CI 1.1, 1.8, *p* = 0.02). Mutant tumors were more common on the head and neck or upper limb compared to the trunk (OR = 5.7 95%, CI 1.6, 20.7 and OR = 4.1 95%, CI 1.1, 15.2 respectively) and were associated with lentigo maligna melanoma (LMM) subtype (OR = 3.8 95%, CI 1.3, 11.4), though solar elastosis scores did not reflect an association with chronic UV exposure, *p* = 0.8 (Table 2). The *RQCD1* P131L mutant tumors were associated with *BRAF* V600K (OR = 3.0 95%CI 1.1, 8.5) but not V600E or *NRAS* codon 61 mutations (Table 3). *RQCD1* mutant melanomas also tended to be more mitotically active and ulcerated compared to wildtype tumors, although this did not reach significance. There were no associations between *RQCD1* mutation status and presence of regression, tumor infiltrating lymphocytes, a history of non-melanoma skin cancer or other markers of UV damage (solar keratosis or a history of blistering sunburn).

**Table 3:** The association of *RQCD1* mutant melanoma with *BRAF* and *NRAS* mutations

	*RQCD1* mutant		*RQCD1* WT				
Mutation	n	%	n	%	OR	95%CI	*p*-value
***BRAF***							
WT	17	59	448	65	1		
V600E	7	24	193	28	1.0	0.4, 2.3	0.9
V600K	5	17	44	6	3.0	1.1, 8.5	0.04
NRAS**							
WT		90		91	1		
Mutant		10		9	0.7	0.2, 2.5	0.6

WT wild type.

** Analysis weighted by sample selection probabilities to account for absence of *NRAS* measurement in some patients.

Despite having thicker tumors, patients with *RQCD1* mutant melanomas were no more likely to present with nodal disease compared to the rest of the cohort (*p* = 0.3), with only 3 patients (10%) presenting with stage III disease at diagnosis (Table 4.). Only one death has occurred in this extended *RQCD1* mutant group. This patient was relatively immune suppressed with melphalan, thalidomide and prednisone for treatment of IgA myeloma and had stable metastatic melanoma disease until one month prior to death.

**Table 4:** Univariate and multivariate logistic regression analyses for mutation status and nodal disease

Mutation	Nodal status		Univariate	Multivariate^
	Positive(%)	Negative(%)	OR [95%CI] *p*	OR [95%CI] *p*
***RQCD1***				
WT	81 (12)	578 (88)	0.9 [0.3, 2.9] 0.8	0.5 [0.1, 1.9] 0.3
Mutant	3 (11)	25 (89)		

WT wild type.

**NRAS weighted to account for missing data in whole cohort.

^ Multivariate analysis included adjustment for thickness, ulceration and mitotic rate.

### Mutational landscape of the CCR4-NOT complex in the TCGA database

The *RQCD1* gene encodes for a core protein of the CCR4-NOT complex (also known as CNOT9, RCD1), which is important in the regulation of gene expression and mRNA degradation. The protein has an Armadillo-like-repeat structure [16] with *in vitro* nucleic acid binding properties and is highly conserved down to c. elegans (Figure 2b). It has been suggested that armadillo repeats mediate protein-protein interactions [17]. The P131L mutation in *RQCD1* sits on the surface of the protein and is positioned at the start of the helix that runs at the bottom of the cleft, which potentially binds to nucleic acids (Figure 2c) [16]. While the substitution of a leucine has a high helix-forming potential, it is much less restricted in the range of phi/psi dihedral angles compared to proline. This could have significant consequences for the CCR4-NOT complex binding to DNA or other proteins.

**Figure 3:**
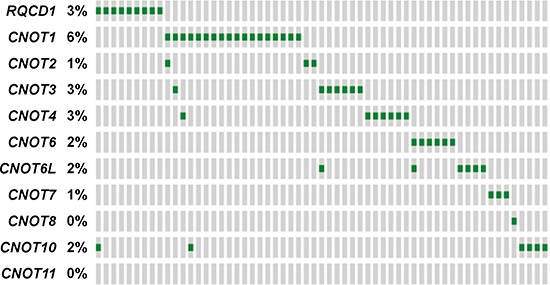
Mutational landscape of the CCR4-NOT complex genes in the TCGA database Mutually exclusive pattern of CCR4-NOT complex gene members based on TCGA mutational data from the subcutaneous melanoma dataset (provisional). Each green rectangle represents the presence of at least one protein altering mutation. A grey rectangle indicates no mutations. The prevalence of a mutation in each gene is shown. Plot extracted from the cBioPortal for cancer genomics.

Other genes that encode for members of the CCR4-NOT complex include *CNOT1*, *CNOT2*, *CNOT3*, *CNOT4*, *CNOT6*, *CNOT6L*, *CNOT7*, *CNOT8*, *CNOT10* and *CNOT11* [18]. Exploration of the mutational status of other members from the TCGA dataset (http://www.cbioportal.org) of subcutaneous melanoma revealed that genes were mutated in a mutually exclusive manner (Figure 3 and Supplementary Figure 1) with 21.2% of cases (*n* = 59) mutated in any one of the complex members. Six patients had the recurrent *RQCD1* P131L mutation, with an additional three patients displaying S87P, N88Y or P131S mutations. All other genes in the CCR4-NOT complex that had mutations had no apparent hotspots.

### *RQCD1* may act as an immunogenic neoantigen

**Figure 4:**
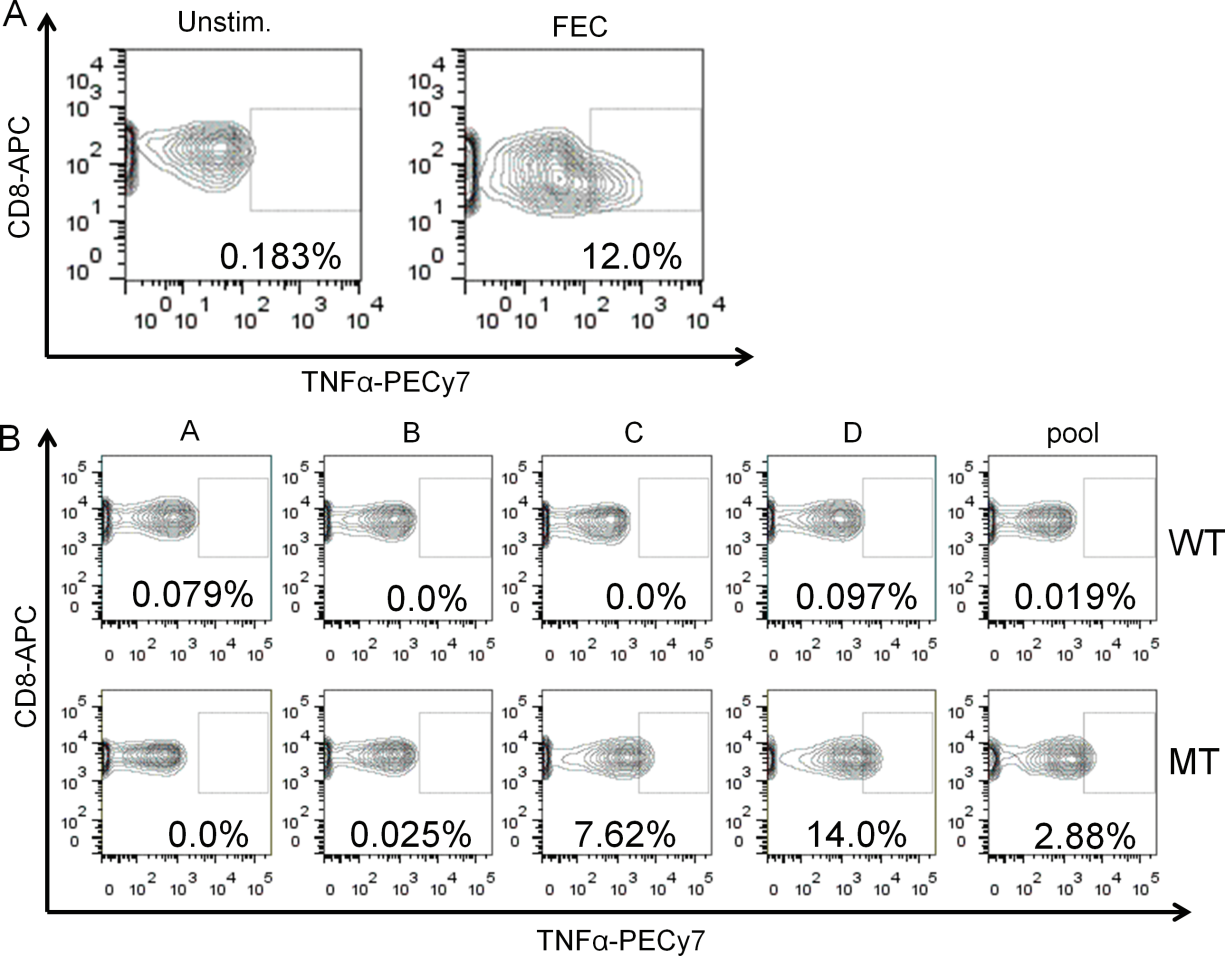
CD8-T cell response to challenge with 13mer peptides representing the mutant or wildtype RQCD1 sequences Percentage of TNFα positive CD8 T cells from a PBMC sample either unstimulated (Unstim., left panel) or treated with an immunogenic peptide pool (FEC) as positive control (right panel). Cells were gated on live and CD3/CD8 double positive cells. Rectangle represents TNFα positive fraction. (B). PBMCs from patient P11076 were stimulated with the indicated peptides (wild type = WT and mutant = MT) alone or as pool and percentage of TNFα positive CD8^+^ T cells evaluated by flow-cytometry. The gate for positivity was set on untreated control from the same patient.

Of the three *RQCD1* P131L positive cases from the discovery set used for exome sequencing, one patient displayed a *BRAF* V600E mutation (LM-MEL-73) and the other two were *BRAF* and *NRAS* wild-type (Figure 1). All three patients shared a common HLA type (HLA-A0201), and *in silico* prediction analysis of the mutant RQCD1 protein suggested strong binding affinity towards HLA-A0201 and HLA-Cw3 of epitopes harboring the mutation (Table 5). While strong MHC binding is thought to often predict immunogenicity, analysis of the mutant versus wildtype peptides using an immunogenicity predicting algorithm (http://tools.iedb.org/immunogenicity) showed no clear evidence for enhanced immune-recognition of the mutant protein (Supplementary Table 2). To address the immunogenic potential and the conflicting predictions for the recurrent *RQCD1* mutation, 13mer peptides with shifted positioning of the exchanged amino acid (Supplementary Table 3) were synthesized and used to stimulate peripheral blood mononuclear cells (PBMCs), where available, from patients (*n* = 13) harboring melanomas with *RQCD1* P131L. In one out of the 13 tested patients we could detect a clear induction of TNFα-positive CD8^+^ T cells using the mutant peptides when compared to their wild type counterparts (Figure 4). The percentages of TNFα positive CD8^+^ T cells rose to 7.62% or 14% for the mutant peptides B and C respectively. Neither of the other patients PBMCs nor a wildtype control sample gave any clear indication of enhanced immunogenicity of the mutant peptide when compared to the wild type. The HLA-status of mutant *RQCD1* patients can be found in Supplementary Table 4 with results for HLA-A and -C alleles, all frequencies were in keeping with the frequencies reported in literature [19].

**Table 5:** Predicted HLA binding scores for wildtype and mutant *RQCD1*

Start residue	Sequence	HLA type	WT Score (BIMAS)	P131L Score (BIMAS)	WT score SYFPEITHI	P131L score SYFPEITHI	WT Affinity (NetMHC)	P131L Affinity (NetMHC)
130	RP/LFEYLRLT	A0201	Not in top 20 predicted binders (i.e. no score)	52.002	12	22	14874 nM	56 nM
126	VSKTRL/PFEY	A0101			21	21		
131	L/PFEYLRLTS	A0101			13	14		
126	VSKTRL/PFEY	B44	4.5	6.75			24724 nM	24724 nM
124	HTVSKTRL/PF	B44	4.5	4.5			20611 nM	20611 nM
129	TRL/PFEYLRL	Cw0301	20	200				
124	HTVSKTRL/PF	B62					156 nM	2929 nM

For the NetMHC analysis, strong binding is < 50 nM, with weak binding between 50 nM to 500 nM. Shown here is predicted binding for epitopes incorporating the 131 amino acid only.

## DISCUSSION

The large scale exome sequencing of multiple tumor types performed by international consortia (TCGA and ICGC) and multiple smaller groups have demonstrated a wide range of mutation loads spanning four orders of magnitude within tumors. For example, pediatric and hematologic malignancies have around 10 mutations on average while lung carcinomas and melanomas have loads typically in the thousands to tens of thousands [19]. The high mutational load in melanoma has been clearly attributed to UV exposure and is characterized by a distinct molecular signature of damage, i.e. C > T nucleotide changes at dipyrimidine residues. While most UV induced mutations in melanoma are thought to not provide a selective advantage to tumor development or growth, there is an increasing volume of evidence that some of these genetic changes are important in the development or progression of melanoma.

In this study, we have identified a recurrent missense mutation in the *RQCD1* gene that is consistent with a UV induced signature. Comparisons between the mutation and clinico-pathological variables showed an association with chronic sun damage such as a LMM subtype [20], a head and neck/upper limb anatomical location and with a *BRAF* V600K mutation status [21]. Associations between UV-based mutations and clinical factors have been described previous for *TERT*, with two hotspot mutations in the *TERT* promoter associated with poor prognostic factors such as Breslow thickness and ulceration [22]. In a similar manner, *RQCD1* mutant tumors were also significantly associated with increased Breslow thickness.

The functional role of the RQCD1 protein remains mostly unknown at present with most studies focusing on its involvement as a core protein of the CCR4-NOT complex, regulating gene expression through mRNA degradation [23]. It was originally identified as a transcriptional cofactor that mediates retinoic acid-induced cell differentiation of the mouse teratocarcinoma cell line F9 [24]. Limited biological studies have implicated RQCD1 in AKT activation [23] and cell proliferation [23]. This, together with the increased Breslow thickness in *RQCD1* mutant tumors supports the idea that the mutant RQCD1 protein may confer a tumor growth advantage.

The protein has an Armadillo-like-repeat structure with *in vitro* nucleic acid binding properties and is highly conserved, indicating that the P131L is an important functional hotspot. Recently, the CCR4-NOT complex has been described as a major effector in miRNA-mediated gene silencing through binding to miRNA targets [12]. Central to this, RQCD1 plays an important structural role in binding to the CNOT1 scaffold protein and interactions with tryptophan (W)-containing motifs in TNRC6/GW182 proteins [25]. The mutually exclusive pattern of mutations in this CCR4-NOT complex in 20% of melanoma patients illustrates that all members of the complex could play an important role in tumor biology. While the functional significance of the mutation has not been investigated in this study, specific mRNA targets affected by the *RQCD1* mutation and the role that other CNOT complexes play in melanoma biology will need to be explored in the future.

With the exception of renal cancer, high mutation load tumors such as melanoma and non-small cell lung adenocarcinoma have been implicated in good response to immunotherapy treatment due to a large repertoire of neoantigens produced from missense mutations. Evidence for this notion comes from studies showing positive T-cell immunoreactivity to predicted mutated peptides from exome sequencing data [26–28]. Moreover, van Rooij and colleagues demonstrated a dominant T-cell response against a mutant epitope that increased strongly after ipilimumab treatment in a melanoma patient [29]. The possibility that the recurrent *RQCD1* P131L mutation is a gain-of-function mutation and the strong binding score for several HLA-types when compared to wildtype gave rise to the possibility that the mutation represents a functional neoantigen. This would represent the most favourable antigen for therapeutic interventions, as tumor-escape due to antigen loss would be unlikely [30]. While this seems to be somewhat counterintuitive, several examples of tumor-promoting mutations that are immunogenic have been described [31, 32]. However, predicted strong HLA-binding does not always translate into *in vitro* immunogenicity [33] and in our small patient cohort just 1 out of 13 patients showed an immunogenic signal for the mutation. As we performed HLA-typing for HLA-A and –C only and our patient cohort was too small to give a true reflection of all possible HLA-types, we can for the moment, not pinpoint the HLA-type that presented the peptide, nor can we conclusively identify the minimal epitope needed.

In conclusion, we have identified a recurrent synonymous somatic mutation in *RQCD1* gene, suggesting that this mutation is being selected for during tumor development. The associations with head and neck and upper limb location, LMM subtype and concurrent *BRAF* V600K mutations are in keeping with the molecular profile of a UV-induced alteration. The *RQCD1* mutation hotspot P131L may be a potent epitope that can elicit a host immune response. While this study has described some of the characteristics associated with the mutation, future studies will be required to test its oncogenic potential and its function in melanoma biology. To determine the role that mutant *RQCD1* plays in interactions with the immune system, additional and more detailed studies in larger patient cohorts are warranted.

## METHODS

### Metastatic melanoma cell lines and patient samples

For the discovery cohort, metastatic cell lines were established from patient material with methods previously described [13, 34]. For the validation cohort, DNA was collected from primary melanoma biopsies from patients enrolled in the Melbourne Melanoma Project (http://www.melbournemelanomaproject.com). Informed consent was obtained for patients in this study. All patients have clinical and histological information collected and are routinely tested for *BRAF* and *NRAS* mutations. Approval for the study was obtained from the human research ethics committees at the Peter MacCallum Cancer Centre.

### Exome sequencing

One μg of DNA was sheared to approximately 200 bp by sonication (Covaris). Exome enrichment was performed using the NimbleGen EZ Exome Library v2.0 kit according to recommended protocols. Sequencing was performed on an Illumina HiSeq2000 instrument. Samples were loaded in an indexed pool of 3 samples per lane, and an average coverage of 141 × was achieved across all samples. Library preparation and sequencing information for each sample is provided in Supplementary Table 1.

### Sequencing alignment and variant calling

Sequence reads were aligned to the human genome (hg19 assembly) using the Burrows–Wheeler Aligner (BWA) program [35]. Local realignment around indels and base quality score recalibration were performed using the Genome Analysis Tool Kit (GATK) [36] software, and duplicate reads removed using Picard [37]. Single nucleotide variants (SNVs) and indels were identified using the GATK Unified Genotyper, Somatic Indel Detector [38] and MuTect (Broad Institute) [39]. Variants were annotated with information from Ensembl [40] Release 64 using the Ensembl Perl Application Program Interface including SNP Effect Predictor.

### Candidate variant identification

Variants were first filtered for confident calls using a quality score cutoff of ≥ 30 and a read depth of ≥ 20. Next, variants were filtered to include only somatic mutations, located in canonical transcripts (the most prevalent transcript as detailed by the UniProt Knowledgebase), with bidirectional read support, and mutations predicted to be potentially deleterious (mutations which potentially change the coding of a protein i.e. non-synonymous, splice site, indels, stop codon lost and stop codon gain mutations).

### High resolution melting analysis and sanger sequencing

PCR and HRM were performed using the LightCycler 480 (Roche Diagnostics). The primer sequences used were 5′-TGCACACTGTCAGCAAAACACG-3′ and 5′-AAACACCCAACTTAGACTACTACT-3′, giving an amplicon size of 127 bp. The reaction mixture included 1x PCR buffer, 2.5 μM MgCl_2_, 200 nM of each primer, 200 μM of dNTPs, 5 μM of SYTO 9 (Invitrogen, Carlsbad, CA), 0.5U of HotStarTaq polymerase (Qiagen), 10 ng DNA and PCR grade water in a total volume of 10 μl. PCR conditions included an activation step of 15 min at 95°C followed by 55 cycles of 95°C for 10 sec, annealing for 10 sec comprising 10 cycles of a touchdown from 65°C to 55°C at 1°C/cycle followed by 35 cycles at 55°C, and extension at 72°C for 30 sec. All analyses were performed in duplicate. At least three different normal controls were included in each run. A positive control sample (LM-MEL-62) was included for each run. Samples showing an aberrant melt profile compared to normal controls via HRM were directly sequenced from a 1/10 dilution of the HRM product using the BigDye Terminator v3.1 cycle sequencing kit (Applied Biosystems, Foster City, CA) according to the manufacturer's instructions.

### HLA typing and immunological studies

HLA binding epitopes within the *RQCD1* sequence were predicted for mutant cell lines using BIMAS (http://www-bimas.cit.nih.gov/molbio/hla_bind/), Syfpeithi (http://www.syfpeithi.de/Scripts/MHCServer.dll/EpitopePrediction.htm) and NetMHC (http://www.cbs.dtu.dk/services/NetMHC/). Blood samples were obtained from 13 patients (Victorian Cancer Biobank, *n* = 11 and the Ludwig Institute for Cancer Research, *n* = 2) with samples sent to the Red Cross for HLA typing.

### T cell activation experiments

We designed overlapping 13mer peptides spanning the region of RQCD1 containing the P131L mutation, with either P (wild type (WT) peptides) or L (mutant (MT) peptides) at position 131 (Supplementary Table 3) (Mimotopes). Peptides were added to patient PBMC either as WT or MT peptide pools or as individual peptides at a final concentration of 1 μg/ml. A peptide mix consisting of immunogenic epitopes from Flu, EBV, and CMV, (FEC peptide pool) was added to one sample/patient as a positive control for T cell activation [41]. Samples were incubated for 10 days in TCRPMI (RPMI 1640, 20 mM HEPES, 60 mg/L penicillin, 12.5 mg/L streptomycin, 2 mM L-glutamine, 1% non-essential amino acids, 10% human AB serum) supplemented with 25 IU/ml IL2. IL2 was replenished during the culture period on days 3 and 6. Following the incubation period, cells were restimulated with the same peptide(s) used in the original stimulus for 4–6 hrs in presence of 10 μg/ml brefeldin A. Cells were then washed and stained with anti-CD3-FITC and anti-CD8-APC mAbs (Beckon Dickinson) for 20 minutes at 4°C, fixed with 1% paraformaldehyde, washed, and stained with anti-TNFα-PeCy7 mAb (eBiosciences) in a 0.25% saponin buffer. Samples were analysed by flow cytometry and data analysed using FlowJo software (version 3.4; Tree Star, SanCarlos, CA).

### Statistical analysis

Univariate logistic regression was used to assess associations between *RQCD1* mutation status and other variables. Univariate and multivariate logistic regression models for the risk of presentation with nodal disease were used for analyses of associations with mutation status with adjustment for known prognostic variables in the multivariate models. As thickness and mitotic rate data were skewed, values were log (base 2) transformed to follow approximately normal distributions. A *p*-value ≤ 0.05 was considered significant. As not all samples were tested for *NRAS* mutations, weights were applied in the relevant analyses and calculated as the inverse probability of selection for testing. All analyses were performed using Stata statistical software version 12.1.

## SUPPLEMENTARY FIGURE AND TABLES




