# Supplementary material for: Whole exome sequencing identifies a recurrent RQCD1 P131L mutation in cutaneous melanoma
Source: Oncotarget. 2014 Dec 6;6(2):1115–27. doi: 10.18632/oncotarget.2747 (PMC4359221; doi:10.18632/oncotarget.2747)
Supplement: Supplementary file 1 [file oncotarget-06-1115-s001.pdf]

SUPPLEMENTARY FIGURE AND TABLES

Gene	CNOT1	RQCD1	CNOT3	CNOT4	CNOT10	CNOT6	CNOT6L	CNOT2	CNOT7	CNOT8	CNOT1
CNOT1	---	0.543724	0.376319	0.376319	0.332294	0.667706	0.667706	0.181941	0.818059	0.935484	1
RQCD1		---	0.792884	0.792884	0.180086	0.819914	0.819914	0.905987	0.905987	0.967742	1
CNOT3			---	0.835407	0.857392	0.857392	0.142608	0.926346	0.926346	0.97491	1
CNOT4				---	0.857392	0.857392	0.857392	0.926346	0.926346	0.97491	1
CNOT10					---	0.876659	0.876659	0.936639	0.936639	0.978495	1
CNOT6						---	0.123341	0.936639	0.936639	0.978495	1
CNOT6L							---	0.936639	0.936639	0.978495	1
CNOT2								---	0.967974	0.989247	1
CNOT7									---	0.989247	1
CNOT8										---	1
CNOT11											---

Legend
Strong tendency towards mutual exclusivity (0 < Odds Ratio < 0.1)
Some tendency towards mutual exclusivity (0.1 < Odds Ratio < 0.5)
No association (0.5 < Odds Ratio < 2)
Tendency toward co-occurrence (2 < Odds Ratio < 10)
Strong tendendency towards co-occurrence (Odds Ratio > 10)
No events recorded for one or both genes

Supplementary Figure 1: Mutually exclusive pattern of CCR4-NOT complex gene members based on TCGA mutational data from the subcutaneous melanoma dataset (provisional). Table extracted from the cBioPortal for cancer genomics (Cerami et al. *Cancer Discovery*. May 2012 2; 401).

Supplementary Table 1: Exome sequencing performance metrics

Supplementary Table 2: Analysis of immunogenicity of mutant *RQCD1* through IEDB

Peptide	Length	Score
VSKTRLFEYLRLT	13	0.24332
VSKTRPFEYLRLT	13	0.24332
TRLFEYLRLTSLG	13	0.12734
TRPFEYLRLTSLG	13	0.12734
LHTVSKTRLFEYL	13	−0.047
LHTVSKTRPFEYL	13	−0.047
YPFLHTVSKTRLF	13	−0.161
YPFLHTVSKTRPF	13	−0.161

Supplementary Table 3: RQCD1 peptides

13 mers (individually and in mt/wt pools)

	Mutant	Wild type
A	YPFLHTVSKTRLF	YPFLHTVSKTR**P**F
B	LHTVSKTRLFEYL	LHTVSKTR**P**FEYL
C	VSKTRLFEYLRLT	VSKTR**P**FEYLRLT
D	TRLFEYLRLTSLG	TR**P**FEYLRLTSLG

Supplementary Table 4: Patient samples with HLA-typing results

Patient ID	HLA-A	HLA-C
P11076	0301, 124	03, 07
P12754	0201, 3303	0401, 0501
P13488	0201	05, 06
P13719	0201, 1101	304, 0602
P14034	0201, 2501	05, 08
P14322	0201, 0301	0304, 0702
P14416	0206, 2601	0303, 1203
P14600	0101	7
P15097	2902	702
P16021	1101, 3002	0202, 0401
P16036	0101, 0301	0102, 0701
LUD P1	02, 24	5, 7
LUD P2	02, 26	0102, 0303
